# Holt-Laury as a gumball machine

**DOI:** 10.1371/journal.pone.0320576

**Published:** 2025-04-10

**Authors:** Mónica Vasco, María J Vázquez-De Francisco

**Affiliations:** 1 *LoyolaBehLab*, Universidad Loyola Andalucía, Córdoba, Spain; 2 ETEAFoundation-Development Institute, Universidad Loyola Andalucía, Córdoba, Spain; Paris School of Economics, FRANCE

## Abstract

The Holt and Laury [1] task is arguably the most widely employed method for eliciting individual risk attitudes in economics. However, although around 15% of standard experimental subjects fail to complete the task consistently, this fraction increases to an alarming 60-70% in the case of children, teenagers, and other non-standard subject pools. This paper explores extensively a visual version of the Holt and Laury task, where subjects choose six times between two gumball machines. Using a sample of 4,972 adolescents, we find that the *Gumball Machine* task decreases the presence of inconsistencies substantially to 20%. Cognitive skills appear to be a strong predictor of consistency and risk preferences, but gender differences are not found.

## 1 Introduction

Since risk aversion is a fundamental concept in decision-making under uncertainty, it has received considerable attention in economics at least since Cramer [2] and Bernoulli [3]. The seminal papers of Pratt [4] and Arrow [5] provide influential definitions and frameworks to quantify and measure risk aversion. Attitudes toward risk have become more prominent empirically since the 80’s and 90’s with the development of experimental techniques allowing for a simple and systematic elicitation of individual risk attitudes [6]. Nevertheless, risk-aversion elicitation generates certain methodological issues [7].

Arguably, the most commonly employed experimental method to elicit individual risk preferences is the multiple price list (MPL, hereafter) by Holt and Laury [1], where ten pairwise choices between lotteries with fixed payoffs but changing associated probabilities are presented to subjects. The distinction between different levels of risk-seeking behavior is an advantage of the Holt-Laury task (HL, hereafter) over other tasks, such as Gneezy and Potters [8] and Eckel and Grossman [9].

The main issue with the HL is related to the complexity of the task and the cognitive demands required, and therefore to the cognitive abilities of the subjects [10]. Indeed, one particular problem is that non-standard subject pools (e.g. children, teenagers, people with low levels of education among others) struggle to fill the task consistently: while most of the experiments with standard experimental subject pools (e.g. adult volunteers, university students) report that around 15% fail to complete the HL task consistently [1,11–14], this number becomes alarming with non-standard participants [15–17]. Since inconsistent subjects might systematically differ in terms of cognitive abilities, literacy, and other characteristics, the common practice of discarding their answers from the data might biashe elicited distributions of risk preferences. In addition, power is reduced at higher inconsistency rates, as inconsistent subjects are excluded [1,7,14,18–20]. In line with these arguments, Charness et al. [21] document that both the complexity and the structure of the employed mechanism affect the elicited risk preferences (see also Andersson et al. [22]). Moreover, Amador-Hidalgo et al. [23] show that lower-ability subjects are more likely to be inconsistent. Indeed, there is considerable evidence that individuals with lower cognitive abilities tend to exhibit inconsistent decision-making behavior in risk-taking tasks [24,25]. Although intuitive tasks, such as BART (Balloon Analogue Risk Task) proposed by Lejuez et al. [26], have been developed from a very different approach taken by psychologists, they do not provide comparable data and the task is slightly difficult to implement in the field [7]. Besides, Crosetto and Filippin [27] proposed BRET (Bomb Risk Elicitation Task) as an alternative measure of individual risk preferences. However, it seems to generate results that are not comparable with HL [28].

Given that many human preferences and behaviors emerge early in life, comprehending the behavior of children and adolescents is essential for understanding adult behavior. Consequently, numerous experimental studies focusing on children and adolescents have developed (e.g., [20,29,30]). Regarding risk attitudes, Sutter, Zoller, and Glätzle-Rützler [31] suggests that the results of the studies analyzed differ substantially (e.g. Sutter et al. [32–34]). Moreover, Defoe et al. [35] demonstrate that whether adolescents exhibit riskier decision-making than adults largely depends on the task used to assess these preferences. Experimental economists thus need a task that elicits children’s risk attitudes reliably and consistently.

In this paper, we explore exhaustively a new risk-elicitation task, the *Gumball Machine* introduced by Alfonso et al. [36] (*gumball*, henceforth). This task is a variation of the HL task, modifying two main features: it is shorter and, more importantly, visual. In Alfonso et al. [36], three waves of adolescents are used to discuss different experimental designs and methods of data collection in schools. Regarding individual risk attitudes elicitation, in its first wave HL is used (715 adolescents), while in its third wave, *gumball* is introduced with 959 observations. We elicit now the risk preferences in a sample of 4,972 adolescents using the *gumball* task in order to: *i*) compare the *gumball* outcomes in terms of inconsistencies and risk-taking *types* (averse, neutral, lover) with other adolescents who performed the original HL, *ii*) explore the determinants of *consistency* and, *iii*) explore potential *order* effects – in terms of the position of the task within the experiment and the position of the balls within the machine (*design*).

We find that the *gumball* increases the consistency among adolescents to nearly 80%, a number comparable with standard experimental subjects but dramatically higher compared to the results of adolescents using the original HL list format. The comparative analysis was carried out with a sample of 715 adolescents included in the first wave of Alfonso et al. [36], using HL plus an eleventh decision (see Table A2 in S1 Appendix). We also observe that the distribution of risk attitudes slightly shifts towards risk neutrality, a result consistent with Bosch-Domènech and Silvestre [37], who analyze reduced versions of HL, and Estepa-Mohedano and Espinosa [19], who added a different visual element to the task (such as bills, beans, and pie chart). Concerning individual heterogeneity, we find no gender effects, but we observe that older teenagers and those with higher cognitive abilities are more likely to exhibit consistent behavior. For consistent subjects, we also find a positive correlation between cognitive abilities and risk aversion. In addition, we find no order effects. Hence, we support the use of the *gumball* task for eliciting individual risk preferences among adolescents.

The paper is organized as follows. The next section introduces the *gumball* task. Section 3 outlines the procedures of the experiment. Section 4 presents the results and the last section concludes.

## 2 The Gumball Machine task

We explore a new elicitation task of attitudes toward risk based on a gumball machine, which retains the core features of HL. In this new task, the classic list of lottery pairs is replaced by pairs of gumball machine figures, where the payoffs are displayed as balls. However, unlike the original HL, this version is shorter. Each subject is asked to make six decisions between options A and B, where option A is safer. In addition, it includes an initial control question, which contains a dominated option. Table 1 provides a summary of the payoffs and the expected values of each decision, as well as the coefficient of relative risk aversion that would make a subject indifferent between A and B for the choice problem, considering that subjects follow a CRRA utility function with the form: $U(x)=\frac{x^{1-r}}{1-r}$.

**Table 1:** Features of the *Gumball Machine* task.

#	A					B					$EV^{A}$	$EV^{B}$		$r^{*}$ (GM)	$r^{*}$ (HL)
	*p*		1–*p*			*p*		1–*p*			()	()			
1	0	10	1	8		0	20	1	2		8	2		*χ*	-
2	0.2	10	0.8	8		0.2	20	0.8	2		8.4	5.6		-0.79	-0.95
3	0.4	10	0.6	8		0.4	20	0.6	2		8.8	9.2		0.09	-0.15
4	0.6	10	0.4	8		0.6	20	0.4	2		9.2	12.8		0.77	0.41
5	0.8	10	0.2	8		0.8	20	0.2	2		9.6	16.4		1.54	0.97
6	1	10	0	8		1	20	0	2		10	20		( *I* + *C* )	*D* ≡ 0

Note: The last column reports the relative risk aversion coefficient in the original HL task [1]. Beware that HL consists of ten decisions and only the five decisions that correspond with the same probabilities are provided. See S2 Appendix for the complete features of the original HL task.

**Fig 1:**
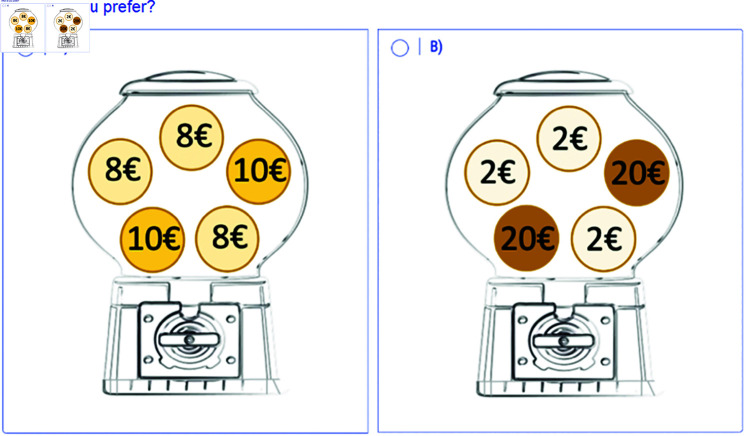
Decision #3 of the *gumball* task.

Fig 1 displays a screen of a decision presented to the subject, which corresponds to decision #3 in Table 1. In every decision, each machine contains five balls with the same probability of coming out (1/5). The six decisions are presented to the subjects one by one on different screens, but in the same order (without randomization). Both options are also fixed, A on the left and B on the right. The complete task is included in S3 Appendix, where Fig A1 displays the original version. In order to test the possible effects of the distribution of the balls within the machine, we ran some sessions in which the balls were placed in different positions (see Fig A2 in S3 Appendix).

Decision-makers can be classified as either *consistent* or *inconsistent* with their decisions. We assume for a *consistent* subject to start by choosing option A and finish by choosing option B, thus switching from A to B only once. In contrast, following the literature, we consider a subject to exhibit a *inconsistent* behavior in this task if she switches back from B to A, selects option B in the first decision (#1), or selects option A in the sixth decision (#6) (both decision problems under certainty) [10,21]. Indeed, we do not measure the number of total inconsistencies. Instead, we use it to nested classify subjects considered as inconsistent. We propose a classification for individuals who appear to exhibit inconsistent behavior:

- *Lack of Understanding* (*LoU*): subjects who select B in the first decision (see screen 1 in Fig A1 and A2 in S3 Appendix). Although there is no uncertainty in this decision, the option with the lowest expected value is chosen. If a subject chooses a dominated option in the first decision, we can assume that she started the task without fully understanding it. Therefore, they are considered as inconsistent subjects, regardless of their subsequent decisions.
- *Switch Back* (*SB*): those who do not satisfy LoU but switch back from B to A in any of the following decisions. That is, once they move to option B, they return to option A.
- *Lack of Attention* (*LoA*): those who neither satisfy LoU nor switch back along the decisions but select A in the sixth decision (see screen 6 in Fig A1 and Fig A2 in S3 Appendix). As in the first type - *LoU*, this decision is under certainty and option A is dominated. Nevertheless, we consider that the subjects may have lost their concentration (or attention) at this stage of the task since they were displaying consistent behavior in the previous decisions.

Risk attitudes are determined by how many safe choices are made [1]. It must be considered that decision number *S* (Fig 1) is critical because the expected values of decisions A and B are fairly similar in both cases ($EV^{A}$ = 8.8 and $EV^{B}$ = 9.2) and require substantial computation. If a subject decides to select A in this decision, she would be classified as risk-averse, while if she selects B, she would be classified as risk-neutral, as long as she had chosen A in the previous decision. Thus, *consistent* subjects can be classified into three risk levels based on their switch point:

- *Risk-averse* individuals: select A three times or more: AAABBB, AAAABB or AAAAAB.
- *Risk-neutral* individuals: select A two times: AABBBB.
- *Risk-loving* individuals: select A once: ABBBBB.

This specification implies risk neutrality (risk-prone people included) for *I*, risk aversion for *C*, and risk seeking for *R*. However, these coefficients differ from the original HL, where the risk-neutral range is  − 0 . 15 > *r* > 0 . 15 (see S2 Appendix).

## 3 Experimental Procedures

The sample was collected between October 1, 2021, and January 27, 2023, as part of a larger project involving adolescents [38]. The entire experiment was approved by the Ethical Committee of Loyola University Andalusia (No. 20211216, 20200709 and 20190318) and parental consents were obtained in conformity with regulations protecting minors (see Vasco et al. [39] for more details on data collection). We implemented the task using a sample of 4,972 adolescents aged 11 to 17 years, from 22 high schools in Spain. Of these, 4,787 completed the risk task, and only 4,694 completed the entire survey. A detailed summary of the sample is provided in S3 Appendix. All participants completed the experiment in class using a tailored platform to run online web-based experiments. It was conducted in supervised sessions included in their regular scholar schedule, minimizing self-selection effects [31,32]. In experiments involving children and adolescents, attrition must be considered, as opposed to those conducted in the laboratory. They might be too young to pay attention and complete all the tasks [20].

The entire experiment consisted of the elicitation of class networks, time and risk preferences, and cognitive abilities. In order to check potential order effects, we ran additional sessions with a different order (networks-risk-time-abilities). The risk task, as previously mentioned and central to this paper, involved six sequential decisions presented one at a time on separate screens, always in the same predetermined order. Subjects completed the tasks using their mobile phones or tablets. In addition, hypothetical payments are provided as incentives. Alfonso et al. [36] test the impact of mobile phone use among adolescents while Brañas-Garza et al. [40] compare results using real and hypothetical incentives including a reduced version of HL. They find no significant differences, as well as, Kühberger, Schulte-Mecklenbeck, and Perner [41] report that both real and hypothetical decisions lead to similar choice patterns. Additionally, Chuang and Schechter [42] present results from risk studies using hypothetical incentives (see also Read [43] Beattie and Loomes [44] and Horn and Freund [45]).

## 4 Results

### 4.1 Types of inconsistency

**Fig 2:**
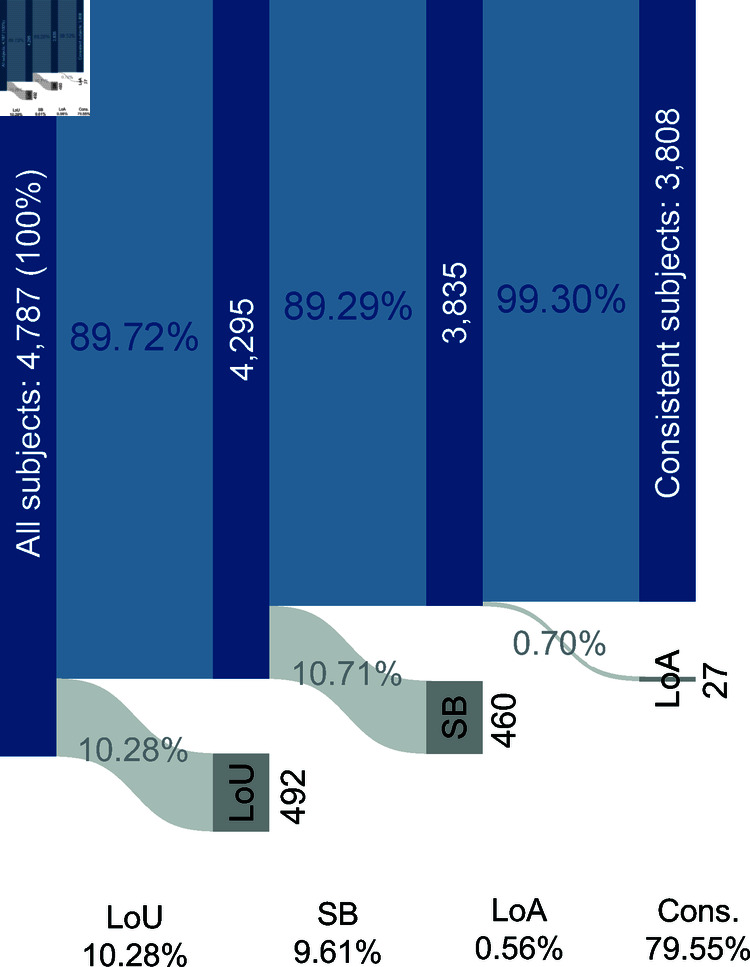
Distribution of consistent and inconsistent subjects.

Fig 2 shows the number and fractions of observations that are inconsistent following our classification and those that are consistent. Our task achieves a higher consistency ratio, reaching values close to those typically observed in adult samples [1,11–14]. This result contrasts previous studies using the list format with children and adolescents, where the consistency ratio was notably lower (e.g., Alfonso et al. [36] reported only 34.83% consistency, see S1 Appendix for further details).

Not only it is observed a high percentage of consistent responses, but also, the level of attention is high, almost all of those who show consistent behavior up to decision 5 maintain their level of attention (99.30%). In addition, by decreasing the cases of *LoU*, we can confirm the advantage of using a visual task in terms of understanding among young people. The effect is combined with the advantage of a shorter task for remaining inconsistencies types. Hence, the *gumball* task greatly reduces all types of inconsistency.

**Determinants of consistent behavior.** We also analyze the potential determinants of consistent behavior. Table 2 presents the estimated results of the regression models (Logit) in *consistency*, which takes a value of 1 if the subject behaves consistently or 0 if the subject is classified in any of the three categories of inconsistent behavior (*LoU*, *SB* or *LoA*). The models include school fixed effects, as well as controlling for *female*, *age*, and several cognitive skills items that are explained below.

**Table 2:** Regression on consistency.

	(1)	(2)	(3)
	*consistency*	*consistency*	*consistency*
*female*	-0.134*	-0.080	-0.078
	(0.074)	(0.075)	(0.075)
*age*	0.138***	0.117***	0.105***
	(0.033)	(0.034)	(0.034)
*GPA*	0.605***	0.589***	0.515***
	(0.109)	(0.109)	(0.111)
*CRT*	0.755***		0.593***
	(0.143)		(0.148)
*finance*		0.941***	0.814***
		(0.151)	(0.155)
*order*	-0.009	0.054	0.014
	(0.322)	(0.327)	(0.326)
*design*	-0.294	-0.237	-0.228
	(0.307)	(0.310)	(0.309)
Constant	-0.925**	-0.595	-0.635
	(0.469)	(0.474)	(0.472)
Observations	4,694	4,694	4,694
Pseudo $R^{2}$	0.0350	0.0375	0.0411
VIF	4.18	4.07	4.19

Robust standard errors in parentheses

*** p<0.01, ** p<0.05, * p<0.1

Note: School fixed effects are also considered among the control variables. Potential order effects are controlled by the variables *order* (position of the task within the experiment) and *design* (distribution of the balls inside the machine). Both are dummy variables that take value 1 if they change from the original version.

Firstly, based on the value of pseudo $R^{2}$, we find that model (3) improves by including all the tasks to measure cognitive skills (*r*=0.3, P-value < 0.001). Secondly, a positive effect is observed between consistency and the following cognitive skills of the subjects: *i*) in line with Amador-Hidalgo et al. [23], a more reflective behavior measured by the adapted cognitive reflection test (*CRT*) from Thomson and Oppenheimer [46], increases the consistent behavior; *ii*) students with better financial skills (*finance*; which covers three exercises involving mathematical and financial calculations) are more likely to exhibit consistent behavior; *iii*) and adolescents with higher academic grades (Grade Point Average; *GPA*) tend to be consistent in the *gumball* task. Therefore, cognitive skills are good predictors of consistency. We also observe that consistency seems to improve with age, but we do not find robust differences between genders.

**Fig 3:**
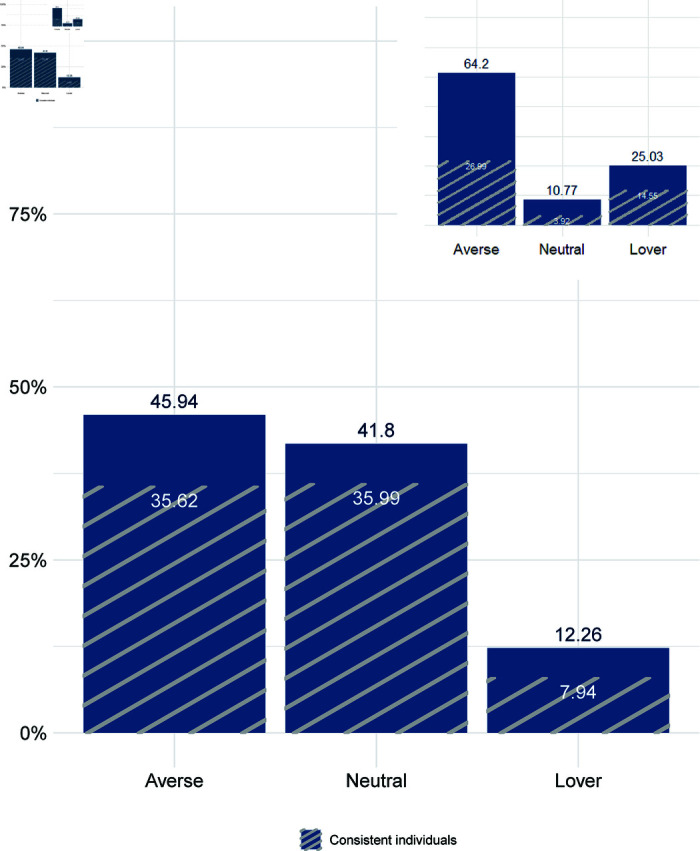
*Gumball*: Distribution of risk preferences. Note: Subjects are assigned according to the number of safe responses, including inconsistent ones as defined by Holt and Laury [1]: 3 or more safe answers is *risk-averse*, 2 is *risk-neutral* and 1 is *risk-lover*. The grid represents the proportion of consistent subjects in each category (79.55% out of 4,787). The upper right sub-plot represents the distribution of the AC-HL sample (for more details see S5 Appendix).

### 4.2 Risk profiles

Fig 3 displays the fractions of risk-averse, risk-neutral, and risk-loving individuals in the sample. The dark color represents consistent subjects.

In line with Bosch-Domènech and Silvestre [37], we observe that the fraction of risk-averse adolescents decreases, shifting towards risk neutrality. If we focus only on consistent subjects, 35.99% show risk-neutral behavior, which is considerably higher than in the original version of the HL task for teenagers. Indeed, the data of AC-HL documents less than 4% of consistent subjects classified as neutral (see Fig A1 in S5 Appendix, for further details). In contrast, the percentage of risk-loving individuals remains similarly distributed. Furthermore, Fig A2 in S5 Appendix, plots the entire distribution of safe choices for both our data and AC-HL sample (transformed into six decisions).

Finally, Table 3 explains the potential determinants of risk aversion only for consistent subjects behavior using the *gumball* task. We perform an OLS regression analysis to estimate the relationship between the number of safe choices (option A) in the six decisions of the *gumball* task. As in the previous models (1), we also control for *female*, *age*, and the cognitive skills variables.

In line with Charness et al. [21], gender does not explain risk aversion. However, it seems to change with age. Older adolescents tend to be more risk-averse. In addition, our results suggest a positive correlation between the number of safe choices and the skills of the subjects which is supported by the literature [23,47]. We observe that *i*) those with more reflective behaviour (*CRT*) tend to show more risk aversion; *ii*) students with better financial skills (*finance*) are more likely to choose safer lotteries; *iii*) and adolescents with higher academic scores (*GPA*) tend to make less risky choices.

**Table 3:** Regressions on the number of safe choices (only consistent subjects).

	(1)	(2)	(3)
	# *safe choices*	# *safe choices*	# *safe choices*
*female*	-0.022	-0.015	-0.012
	(0.029)	(0.029)	(0.029)
*age*	0.032**	0.032**	0.027**
	(0.013)	(0.013)	(0.013)
*GPA*	0.172***	0.186***	0.155***
	(0.042)	(0.042)	(0.043)
*CRT*	0.268***		0.238***
	(0.056)		(0.058)
*finance*		0.183***	0.130**
		(0.054)	(0.055)
*order*	0.179	0.204*	0.185
	(0.118)	(0.119)	(0.119)
*design*	0.034	0.044	0.044
	(0.120)	(0.121)	(0.120)
Constant	1.719***	1.778***	1.770***
	(0.179)	(0.180)	(0.179)
Observations	3,747	3,747	3,747
Adjusted $R^{2}$	0.024	0.021	0.025
VIF	2.68	2.69	2.64

Robust standard errors in parentheses

*** p<0.01, ** p<0.05, * p<0.1

Note: School fixed effects are considered in addition to the control variables. Potential order effects are controlled by the variables *order* (position of the task within the experiment) and *design* (position of the balls inside the machine). Both are dummy variables that take value 1 if order or design changes with respect to the original version.

According to Charness, Gneezy, and Imas [7], it is common to find cases where subjects switch options several times, therefore models that add these errors as a stochastic component are often reported. Because the *gumball* task is shorter we also verify how the effect is using this task as opposed to the original version. We applied a maximum likelihood (ML) structural estimation with the Luce error specification [48–50]. We added to our sample, 715 observations from AC-HL sample that represent adolescents who completed the HL task using a classical list format. Therefore, we created a dummy to estimate the effect of the *gumball* task on the risk aversion coefficient assuming a CRRA utility function. Table 4 reports the results of this model, revealing the *gumball* task decreases the risk aversion coefficient compared to HL.

**Table 4:** The *gumball* effects on the risk aversion coefficient (*r*).

	r
*age*	0.007**
	(0.003)
*CRT*	0.038**
	(0.017)
*gumball*	-0.073***
	(0.017)
Constant	0.869***
	(0.046)
	
Subjects	5,444
Observations	32,665
Robust standard errors in brackets	
*** p<0.01, ** p<0.05, * p<0.1	

### 4.3 Order effects

We test order effects. Two types are considered: *i*) the order in which the task appears in the protocol (*order*); and *ii*) the position of the balls within the machine (*design*). We do not observe a significant impact on either consistency nor on the level of risk aversion in previous results (Tables 1 and 3). In the same vein, there is no significant effect due to variations in the order of implementation of tasks, which would confirm the robustness of the *gumball* task to elicit risk preferences among adolescents. See S6 Appendix, for the results of the estimation of effects on the maximum likelihood estimation of the risk aversion coefficient assuming a CRRA utility function.

## 5 Conclusions

Risk aversion has been measured traditionally as one of the classical variables of economic preferences in the field of experimental and behavioral economics. Although adult populations have been the standard experimental subjects involved in such studies, there is a growing interest in the economic behavior of children and adolescents. This is due to the interest in predicting, explaining and, sometimes, preventing future events related to educational performance, health, or disruptive behaviors. The widely used MPL of Holt and Laury [1] to elicit individual risk preferences, presents some limitations when administered to different ability groups to standard subjects, such as children or rural populations. These groups may have difficulties due to the high computational demands, making it difficult to perform the task consistently. Consequently, high levels of inconsistent behavior are observed, which prevents drawing clear findings in such samples.

This paper contributes to the literature on risk measurement by addressing key limitations of traditional methods when applied to non-standard populations like adolescents. A short and visual method to measure risk aversion especially thought for teenagers is explored: the *Gumball Machine* task. Using this task, we elicited risk preferences from a large sample of adolescents in Spanish high schools. Our results show that the inconsistency level decreases greatly from 65.17% to 20.45%. Particularly, less dominated choices are observed in both the first and the sixth decisions. Visual support therefore seems to help students to understand the task better since with the first decision, regardless of the task length, the reduction of *LoU* behaviors is remarkable (from 20.98% to 10.28%). Likewise, the number of switch backs is reduced, also as might be expected due to the shorter task (from 39.86% to 9.61%).

Furthermore, our findings reveal a significant relationship not only between cognitive abilities and consistency but also between cognitive abilities and risk preferences. In line with the existing literature, which suggests that simpler tasks tend to promote risk neutrality, we observe a similar trend with the *gumball* task (e.g. Bosch-Domènech and Silvestre [37]). Additionally, as stated by Defoe et al. [35], preferences can vary depending on the task used to elicit risk attitudes. In our study, the list format in the HL task appears to lead to more averse decisions among adolescents compared to the risk attitudes observed with the *gumball* task. Hence, our analysis indirectly supports the notion that the original HL task might systematically skew the distribution of elicited risk attitudes. This raises concerns that certain risk attitudes might be underrepresented when inconsistent subjects are excluded from the analysis. This issue could be potentially mitigated by using the *gumball* task.

Although this study provides valuable insights and highlights the importance of designing adaptive tools that recognize cognitive diversity and varying ability levels to elicit risk preferences, one issue has not been yet completely disentangled and deserves further investigation: the individual effect of shortening the task versus displaying it visually. Our results reveal already for the first decision a significant decrease in the number of individuals who do not understand the task (*LoU*), along with an increase in consistent behavior across subjects. This suggests a direct impact of our visual tool. However, when considering the task as a whole, we cannot conclusively attribute the overall reduction in inconsistent behavior to only one of these features - the shortening of the task also plays a role. Future research should explore these two factors separately to better understand their distinct contributions. Additionally, extending this research to other non-standard populations, such as younger children or individuals with diverse cognitive abilities, could offer new insights. Finally, this task offers a promising approach to a gender-neutral method for eliciting risk preferences.

## Supporting information

S1 Appendix. **Samples of adults vs. adolescents** (PDF)

S2 Appendix. **Features of the original HL task** (PDF)

S3 Appendix. **Additional details of the gumball task** (PDF)

S4 Appendix. **Type of inconsistencies** (PDF)

S5 Appendix. **Versions with different decision number** (PDF)

S6 Appendix. **Additional estimation of order effects** (PDF)

## Acknowledgments

We sincerely thank Pablo Brañas and Jaromír Kovářík, for their invaluable guidance and support. Their expertise and dedication were instrumental in the success of this research. We also thank Antonio Alfonso for helping us with the development of the task, Pablo Lomas for leading the field team in high schools and Diego Jorrat and César Mantilla for their valuable feedback for the improvement of the paper.
