# Correction: Holt-Laury as a gumball machine

**DOI:** 10.1371/journal.pone.0357757

**Published:** 2026-09-04

**Authors:** The PLOS One Staff

The second author’s initials appear incorrectly in the citation. The correct citation is: Vasco M, Vázquez-De Francisco, MJ (2025) Holt-Laury as a gumball machine. PLoS ONE 20(4): e0320576. https://doi.org/10.1371/journal.pone.0320576

The Funding statement for this article is incorrect. The correct Funding statement is as follows: This research was supported by the Spanish Ministry of Economy and Competitiveness (PID2021–126892NB-I00), the Spanish Ministry of Education (FPU20/05846), the Andalusian Agency for International Development Cooperation (AACID-0I008/2020), Grant B-SEJ-280-UGR20 funded by the Department of University, Research and Innovation of the Junta de Andalucía and by ERDF A way of making Europe, and the 4th EU-LAC Joint Call STI/2022 (EULAC-2022–166). The funders had no role in study design, data collection and analysis, decision to publish, or preparation of the manuscript.

The publisher apologizes for the errors.
